# Preliminary results of ultrasound-guided laser ablation for unresectable metastases to retroperitoneal and hepatic portal lymph nodes

**DOI:** 10.1186/s12957-016-0917-2

**Published:** 2016-06-23

**Authors:** Yun Mou, Qiyu Zhao, Liyun Zhong, Fen Chen, Tianan Jiang

**Affiliations:** Department of Ultrasound, The First Affiliated Hospital, College of Medicine, Zhejiang University, 79# Qingchun Road, Hangzhou, 310003 China; Division of Hepatobiliary and Pancreatic Surgery, The First Affiliated Hospital, Zhejiang University, 79# Qingchun Road, Hangzhou, 310003 China

**Keywords:** Laser ablation, Metastasis, Hepatic portal lymph node, Retroperitoneal lymph node

## Abstract

**Background:**

Laser ablation with a neodymium-doped yttrium aluminum garnet (Nd:YAG) laser is a minimally invasive approach which is able to achieve a precise tissue necrosis. The study was aimed to assess the feasibility and efficiency of laser ablation in the treatment of retroperitoneal and hepatic portal unresectable metastatic lymph nodes.

**Methods:**

Eight patients including 11 pathologically proven metastatic lymph nodes, 4 in retroperitoneal, 7 in hepatic portal region, were treated by laser ablation. Primary cancers were cholangiocarcinoma (*n* = 4) and hepatocellular carcinoma (*n* = 4). Under sonographic guidance, the laser ablation was performed percutaneously. Follow-up contrast computed tomography or magnetic resonance image was performed.

**Results:**

The treatments were completed in single process in all the patients. No severe complications occurred. Follow-up contrast computed tomography or magnetic resonance imaging at 1 and 3 months showed partial responses in 11 lymph nodes. The local response rate at the 6 month follow-up was 75.0 %. The overall response rate was 62.5 %. Abdominal pain scores decreased significantly in all patients. Tumor marker levels decreased in six patients. The Child-Pugh grade did not change.

**Conclusions:**

The results suggest that sonographically guided laser ablation is technically feasible for the local treatment of unresectable retroperitoneal and hepatic portal lymph nodes from hepatic cancer. Although further study is needed to evaluate its long time efficacy, abdominal pain relief is prominent.

## Background

The common therapies for unresectable metastases are radiotherapy or chemotherapy [1–5]. However, radiotherapy is contraindicated when the radiation dose reaches the upper limit. Some of patients cannot endure the treatment course due to side effects caused by chemotherapy. Image-guided local therapy is a minimally invasive method that can be performed percutaneously. It includes two main technologies: thermotherapy and chemical ablation. Thermotherapy includes radiofrequency ablation, microwave ablation, laser ablation, and high-intensity-focused ultrasound ablation. Chemical ablation includes local injection of ethanol or acetic acid [6–8]. In this technique, the therapeutic electrode or injection needle is inserted into the target region under computed tomography, magnetic resonance, or ultrasound guidance. Radiofrequency ablation and laser ablation has been considered equally effective in treating small hepatocellular carcinoma [6]. Moreover, radiofrequency ablation is considered the first-line treatment in some hepatocellular carcinoma and seems to be comparable to surgical resection in terms of survival outcomes [7, 8]. The advantages of this kind of therapy are local coagulation of malignancies with good toleration in fragile patients and few systemic adverse reactions. Therefore, in the last decade, it has become an effective therapy for some recurrent or unresectable malignancies.

However, the metastases in the hepatic portal or retroperitoneal space are considered to be “critical lesions” and are difficult to be treated by radiofrequency and microwave ablation [9, 10]. The main reason is that blood flow carries away part of the general heat resulting in the “heat-sink” effect [10]. Residual tumor was observed in 100 % of tumors adjacent to the inferior vena cava, in 57 % of tumors adjacent to the portal vein, and in 33 % of tumors adjacent to the hepatic veins [11]. At the same time, with a large diameter of the needle, usually 16 Ga, unintentional injury of the great vessels can produce potentially fatal hemorrhage [9]. Ethanol injection therapy using a 21-Ga needle may decrease the risk in injury of great vessels. However, the liquid can be distributed non-uniformly due to intratumoral septa, which results in incomplete necrosis [8, 12]. Furthermore, a larger number of treatment sessions are required for each lesion, and recurrence or needle track seeding, particularly in tumors greater than 2 cm, is possible [12].

The use of neodymium-doped yttrium aluminum garnet (Nd:YAG) laser ablation provides more accurate thermal field control. It has been used in many conditions such as endobronchial therapy [13], prostate diseases [14, 15], and thyroid tumors [16–18]. Usually, a fiber of only 300 μm in diameter is required. In some studies, laser ablation has also been used in hepatocellular carcinoma [19–21] and considered to be a component of the multimodality treatment of liver metastases from colorectal cancer and neuroendocrine carcinomas [22, 23]. The tumor location does not have a significant negative effect on the effectiveness, safety, or ability to achieve local control of disease. The aim of this study was to apply laser ablation to retroperitoneal and hepatic portal metastatic lymph nodes to assess the safety, feasibility, and efficiency of this treatment in these high-risk areas.

## Methods

### Patients

The study was approved by the Institutional Review Board at the First Affiliated Hospital, College of Medicine, Zhejiang University. The procedures were conducted according to the principles of the Helsinki Declaration. Written informed consent was obtained from the patients for publication and the accompanying images.

From August 2012 to January 2014, eight patients (six men, two women, age range 36–58 years old, mean age 48.0 years old, follow-up period 6 months) underwent sonograghically guided laser ablation. All patients were examined by an interdisciplinary tumor board including surgeon, radiologist, hepatologist, and oncologist. All the patients had a history of surgery for liver cancer and at least one cycle of chemotherapy or radiotherapy. No patients were good candidates for surgical resection of the lymph nodes. Inclusion criteria were (1) metastatic lymph nodes in the retroperitoneum or hepatic portal of liver cancer, (2) a single lymph node less than 5 cm or multiple lymph nodes (no more than three) less than 3 cm, (3) inadequate response to chemotherapy or radiotherapy: continued enlargement of lesion by more than 20 % in spite of sorafenib treatment for 2 months or treatment with maximum dosage of radiotherapy or discontinuation of these treatments because of serious adverse reactions, and (4) target lymph nodes could be revealed by ultrasound. Exclusion criteria were patients with clotting impairment, renal failure, or Child-Pugh grade C cirrhosis. The primary cancers and numbers of cases were cholangiocarcinoma (CCA) 4 and hepatocellular carcinoma (HCC) 4. The target metastatic lymph nodes in the study consisted of four in the retroperitoneum (two in a patient with CCA, two in a patient with HCC)and seven in the hepatic portal region (two in two patients with CCA and five in four patients with HCC). Lymph node size (maximum diameter) ranged from 15 to 31 mm (mean 21.3 mm).

Patients were symptomatic including abdominal pain, weakness, and weight lost. The pain score was assessed before and after treatment according to the criteria described previously [24]: grade 0, no pain; grade 1, mild pain that does not interfere with function; grade 2, moderate pain or pain or analgesics that interferes with function but does not interfere with activities of daily living; grade 3, severe pain or pain or analgesics that severely interferes with activities of daily living; and grade 4, disabling pain.

All lymph nodes were histologically proven to be metastases of primary cancer by sonographically guided biopsy before laser ablation.

### Treatment procedure

**Fig. 1:**
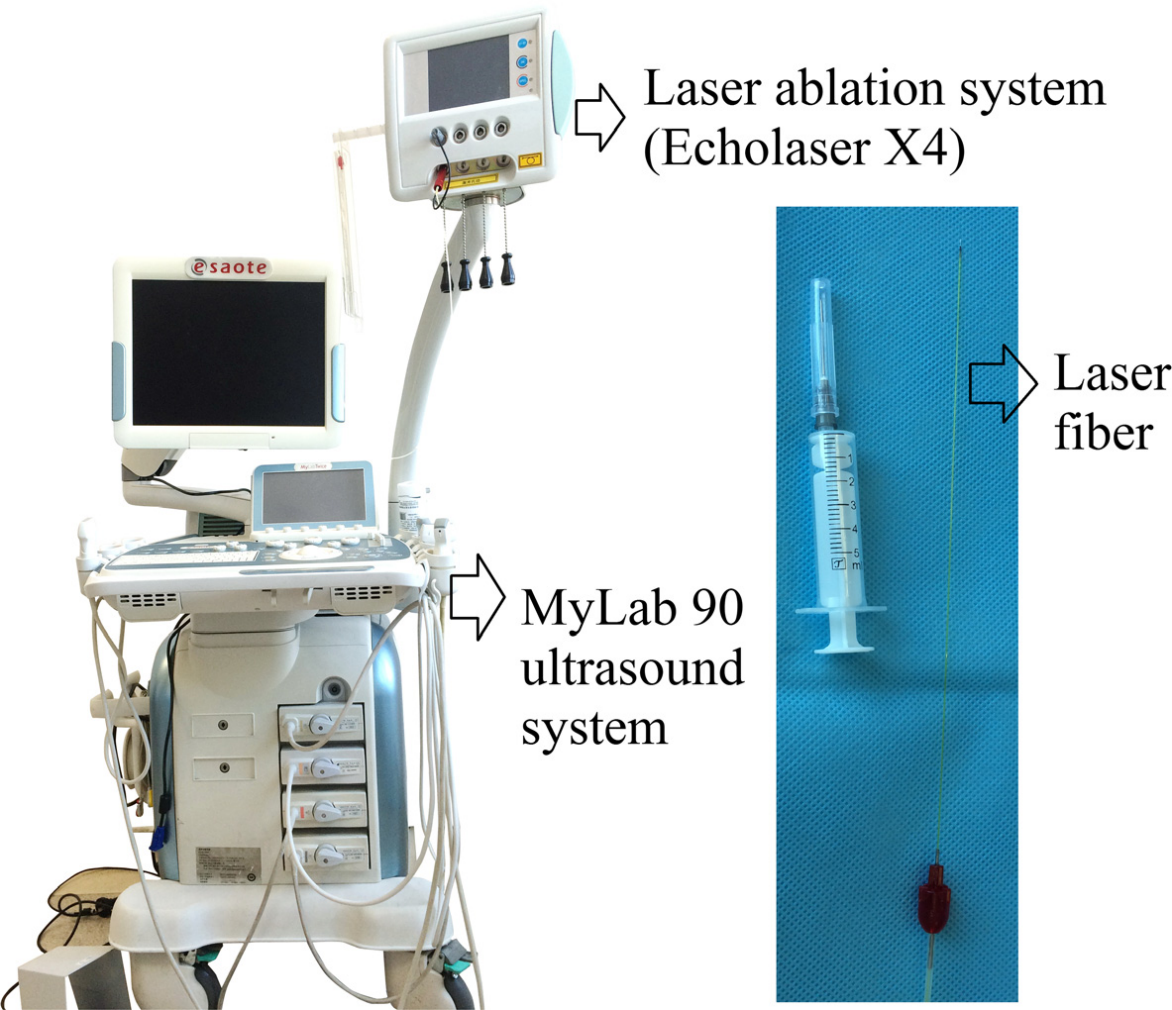
The equipment of MyLab 90 (Esaote, Genova, Italy) together with laser ablation instrument and the picture of laser fiber

Immediately prior to laser ablation, contrast-enhanced ultrasound (CEUS) was used to determine the borders, locations, and sizes of the lymph nodes. The contrast agent, 2.4 mL SonoVue (Bracco, Milan, Italy), was injected intravenously by bolus injection followed by a 10 mL saline flush. The instrument used was a MyLab 90 (Esaote, Genova, Italy) (Fig. 1) with CA541 transducer. The transducer is a convex matrix array probe with a 4–10-MHz frequency. Masses which were enhanced by the contrast agent were considered to be target lymph nodes. The lymph nodes began to be enhanced immediately after aortic enhancement. The duration lasted for about 10–30 s. Each target lymph node size was measured in this arterial phase. The distance between lymph node and inferior vena cava, aorta, and portal vein was measured. The tissue which was in front of the lymph node was carefully observed as well because important structures such as pancreas, bowel, and mesenteric artery should be avoided at the time of puncture.

Laser ablation was performed by a Nd:YAG laser-beam fiber ablation system (Echolaser X4, Elesta s.r.l. Florence, Italy) in a continuous mode, with a wavelength of 1064 nm in which the penetration of light in the infrared spectrum is optimal.

Ultrasound-guided needle insertion into lymph nodes was performed under general anesthesia. The insertion route was carefully determined by ultrasound and color Doppler image to avoid the pancreas, bowel, vessels, or bile ducts. Care was taken to ensure that the tip of a 21-Ga needle was 10 mm away from the distal borderline of the lymph node. After the correct positioning of the tip of the needle, a 300-μm diameter plane-cut quartz optical bare fiber was advanced through the sheath of the needle, and the sheath was withdrawn 10 mm to expose the 10-mm fiber. The output power of laser was set at 3 W, and the duration set for 10 min. Worn fibers automatically block prevent firing of the laser when the system is switched on. If the whole lesion did not become hyperechoic at the end of the first 10 min, in the proximal area of the lymph nodes, the ablation was considered to be insufficient. In those cases, both the fiber and the sheath were withdrawn about 2–5 mm. Additional energy was applied until the lesion became hyperechoic. The total delivered energy was calculated by the equipment.

The number of the laser fibers used was depended by the size of the lymph node. The patient was observed for 30 min after the treatment for changes in vital signs.

### Analysis of therapeutic efficiency

Immediate post-laser ablation, CEUS was performed by the laser device operator. Images were transferred to an ultrasound workstation and evaluated by another doctor with 5 years of experience with CEUS and who was blinded to the protocol. The target area without enhancement by SonoVue was defined as success. The mechanism is based on obstruction of the tumor blood supply by the coagulation necrosis of the target lesion after laser therapy. If there was area in the target lesion that was enhanced by SonoVue, it was defined as a residual area. Then, an additional laser ablation was done immediately and CEUS was performed after that treatment. One month after laser ablation, MRI or contrast-enhanced CT scans were performed to evaluate the efficiency of therapy and then every 3 months. Child-Pugh grade and tumor markers including serum alpha fetoprotein (AFP) levels for HCC and carbohydrate antigen 19-9 (CA19-9) levels for CCA were measured.

Follow-up MRI or CT scans were evaluated by an experienced radiologist who was blinded to the therapy. Therapeutic efficiency was assessed by the criteria for image-guided tumor ablation [25]: (1) complete response (CR) was defined as complete disappearance of the target tumor enhancement detectable by imaging analysis, (2) partial response (PR) was defined as the reduction of the target tumor enhancement by >50 % compared to the pre-procedure value, (3) no change (NC) was defined as a decrease of <50 % or an increase of <25 % in contrast enhancement in the target area compared to the pre-procedure value, and (4) lymph node progression (LP) was defined as an increase of >25 % contrast enhancement or enlargement of the lymph node in the target area. Local response rate (LRR) was calculated as (CR number + PR number)/total number of cases × 100 % [25]. The appearance of new lymph nodes or metastasis elsewhere was also assessed in follow-up. The overall response rate (ORR) was calculated as (CR number + PR number − progression elsewhere)/total number of cases × 100 %.

Statistical analysis: All values were expressed as mean ± SD and statistically analyzed by pared *t* test with SPSS 19.0 software. A *p* value less than 0.05 was defined as statistically significant.

## Results

### Ultrasound guidance and the laser ablation

**Fig. 2:**
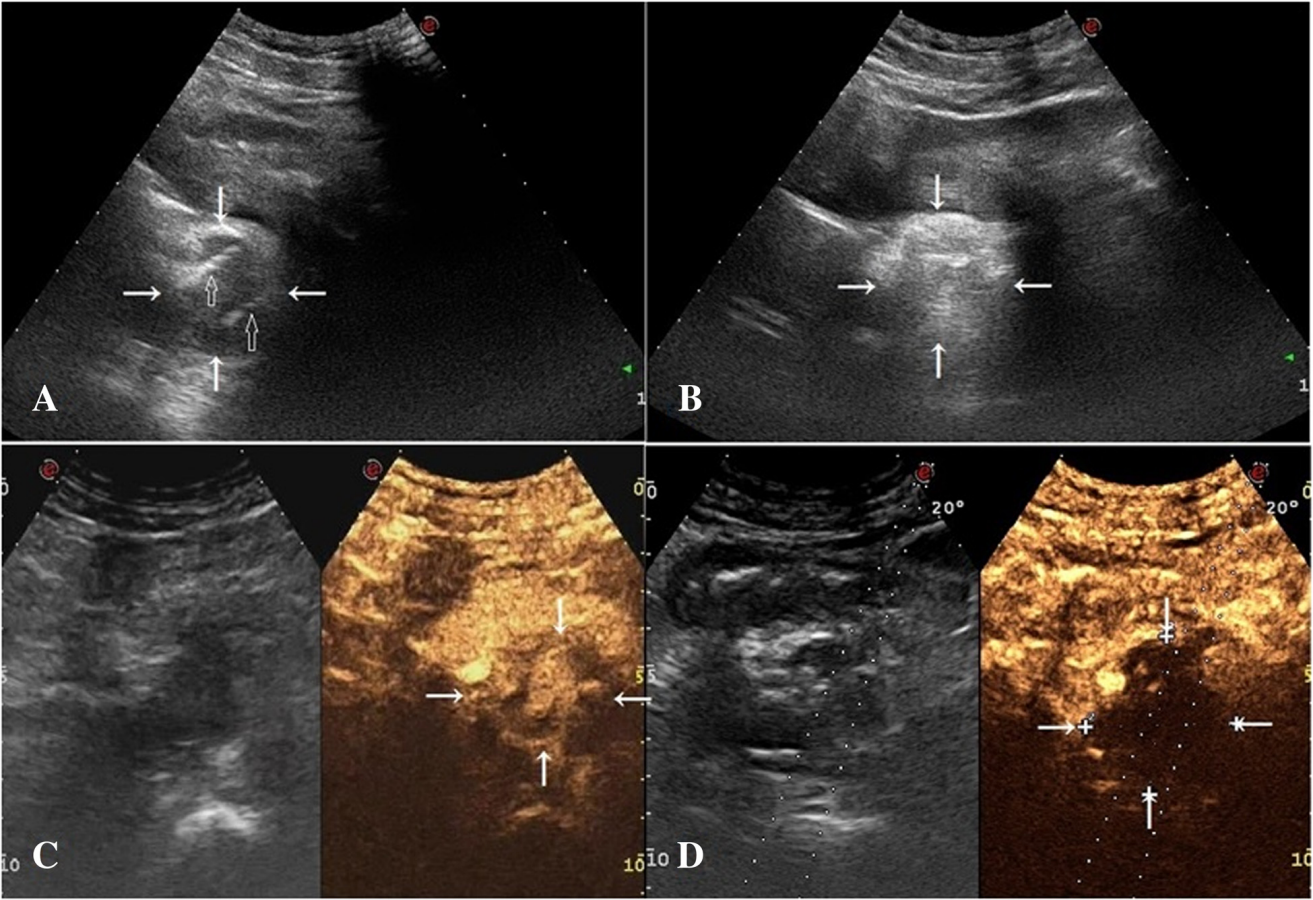
Ultrasound images in case 1 with a retroperitoneal metastatic lymph node. **a** Two optical fibers (*hollow arrow*) were inserted into the hypoechoic nodule (*arrow*). **b** After laser ablation, the nodule was reviewed as a hyperechoic lesion. **c** The nodule was enhanced by SonoVue injection in contrast-enhanced ultrasound before laser ablation. **d** There was no enhancement in the nodule by SonoVue injection immediately after laser ablation

During laser energy application, ultrasound images showed a hyperechoic area around the fiber tip. When the procedure was completed, the whole lesion consisted of a hyperechoic zone (Fig. 2). Laser energy and the number of laser fibers are listed in Table 1. All the lymph nodes were treated by single therapies because no residual enhancement area was found by immediate CEUS.

**Table 1:** Characteristics of patients and their follow-up imaging results

	Gender	Primary cancer	Size (mm)	Location	Number of fibers	Power (w)	Energy (J)	1-month follow-up	3-month follow-up	6-month follow-up	Progression elsewhere
Case 1	m	CCA	31 × 25	Retroperitoneum	2	3	3600	PR	PR	NA	Liver and left kidney progression in 3-month follow-up
Case 2	m	HCC	24 × 12	Hepatic portal	1	3	3000	PR	PR	PR	No
			18 × 15	Hepatic portal	1	3	2700	PR	PR	PR	
Case 3	f	HCC	20 × 14	Hepatic portal	1	3	2700	PR	PR	PR	No
Case 4	m	CCA	31 × 21	Hepatic portal	2	3	3600	PR	PR	PR	Liver progression in 6-month follow-up
Case 5	m	HCC	15 × 12	Hepatic portal	1	3	1800	PR	PR	PR	No
			15 × 11	Hepatic portal	1	3	1800	PR	PR	PR	
Case 6	m	CCA	15 × 12	Hepatic portal	1	3	1800	PR	PR	PR	No
Case 7	f	HCC	18 × 14	Retroperitoneum	1	3	2700	PR	PR	PR	New lymph nodes in retroperitoneum in 6-month follow-up
			28 × 19	Retroperitoneum	2	3	3600	PR	PR	LP	
Case 8	m	CCA	18 × 14	Retroperitoneum	1	3	1800	PR	PR	PR	No

*CCA* cholangiocarcinoma, *HCC* hepatocellular carcinoma, *CR* complete response, *PR* partial response, *LP* lymph node progression, *NA* not available

### Clinical follow-up

**Fig. 3:**
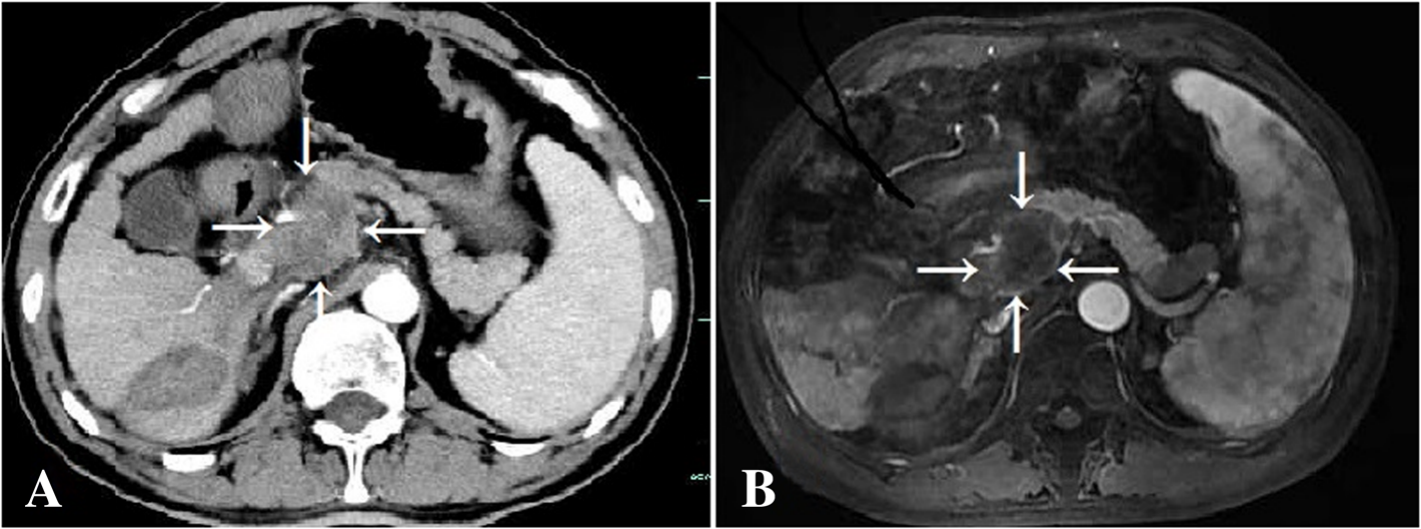
Contrast-enhanced CT pre-treatment and 1 month MRI follow-up images from Case 1, a patient with retroperitoneal metastatic lymph node (*arrow*) treated with laser ablation. **a**: Contrast-enhanced CT showed that a lymph node was enhanced heterogeneously in arterial phase. **b**: There was reduction enhancement of the nodule about 88 % by gadoxetic acid-enhanced MRI in arterial phase indicating a local partial response

One-month follow-up MRI or CT scans showed that all lymph nodes had partial responses with decreased enhancement by about 81–90 % (Fig. 3).

At the 3-month follow-up point, one patient was found to have left kidney metastasis and a liver cancer progression. He died from cerebral hemorrhage 4 months after laser ablation. Seven patients survived by the 6 month follow-up. Six patients had local partial responses. One patient had lymph node progression with two new lymph nodes. One patient had progression in the liver (Table 1). The LRR at the 6 month follow-up was 75.0 %, and the ORR was 62.5 %.

The pain score decreased significantly after treatment in the follow-up period (Table 2).

**Table 2:** Changes in pain scores in the follow-up period

	Pain grade			
Case	Before treatment	1-month follow-up	3-month follow-up	6-month follow-up
1	4	2	2	NA
2	3	1	1	1
3	3	1	1	1
4	4	1	1	1
5	3	1	1	1
6	3	1	1	1
7	4	1	1	2
8	3	1	1	1
Mean ± SD	3.38 ± 0.52	1.12 ± 0.35*	1.12 ± 0.35*	1.14 ± 0.38*
*p* value		0.000	0.000	0.000

*NA* not available

*p < 0.05 vs before treatment

There were no changes in Child-Pugh grades after laser ablation in any patients (Table 3). Serum AFP decreased significantly in 1-month follow-up but not significantly in 3 and 6-month follow-ups. CA19-9 decreased significantly in 3 and 6 month follow-ups but not in 1 month follow-up (Table 3).

**Table 3:** Child-Pugh grade and serum tumor marker follow-up data

	Child-Pugh grade				AFP (ng/mL)				CA19-9 (U/mL)			
Case	Before treatment	1-month follow-up	3-month follow-up	6-month follow-up	Before treatment	1-month follow-up	3-month follow-up	6-month follow-up	Before treatment	1-month follow-up	3-month follow-up	6-month follow-up
1	B	B	B	NA					205.1	195.3	105.4	NA
2	B	B	B	B	151.7	65.2	66.8	79.5				
3	B	B	B	B	97.6	17.6	35.3	75.1				
4	B	B	B	B					545.2	236.1	118.7	302.5
5	A	A	A	A	137.3	54.2	28.6	14.4				
6	B	B	B	B					311.2	142.2	147.4	159.9
7	B	B	B	B	84.2	55.9	78.8	153.6				
8	B	B	B	B					346.6	102.4	98.4	105.8
Mean ± SD					117.70 ± 31.97	48.22 ± 20.98*	52.38 ± 24.24	80.65 ± 56.99	352.02 ± 142.13	169.00 ± 58.73	117.48 ± 21.65*	189.40 ± 101.61*
*p* value						0.015	0.060	0.433		0.066	0.045	0.020

*AFP* alpha fetoprotein protein, *CA19-9* carbohydrate antigen 19-9

*p < 0.05 vs before treatment

### Adverse reaction

There were no major complications detected in the patients during the laser ablation. Low grade to moderate grade fever lasting for 2–4 days after the procedure was detected in seven patients. These resolved with symptomatic treatment.

## Discussion

Laser ablation is one of the thermal therapies that can be used for local control of malignant tumors [19, 20, 24–26]. In the current study, we treated retroperitoneal/hepatic portal lymph nodes by a Nd:YAG laser-beam fiber ablation and found good local response of the malignancies at the 6-month follow-up. To the best of our knowledge, this is the first report in the treatment of metastatic lymph nodes in abdomen by laser ablation.

Retroperitoneal lymph node metastasis is one of the signs of advanced or terminal stage of malignancy. Treatment by ethanol injection and radio frequency ablation has been reported [27–29]. We believe that the main advantage of applying Nd:YAG laser ablation is the use of fine needles, which are less traumatic and can be handled more easily by the operator to reach difficult sites. On the other hand, Nd:YAG lasers with a wavelength of 1064 nm were used because the penetration of light is optimal in the near infrared spectrum. This type of laser has been commonly used in interstitial ablation [6].

CT-guided interventional therapies are widely used. However, the CT scan is limited to cross-sectional anatomy [30]. Ultrasound scanning including color Doppler imaging is feasible to avoid damage to the vessels and organs because it can target the needle in virtually any plane. Therefore, it is possible to choose a safe route, and real-time ultrasound imaging can track the advancement of the needle. In the current study, we used ultrasound to guide the insertion of Nd:YAG laser fibers. No significant puncture-related complications occurred. Moreover, CEUS evaluates ablation effect immediately and decisions on whether additional therapy is required can be made immediately.

In most studies, it has been reported that the treatment of lesions close to the great vessels was more likely to result in residual tumor because of the heat-sink effect of blood flow [7, 9, 10, 29]. In the current study, the lymph nodes either close to the aorta, inferior vena cava, or portal vein had a local response rate of 75.0 % and overall response rate of 62.5 %. Tumor markers decreased significantly in some patients except in two cases with progression elsewhere. These data showed that laser ablation benefits treatment of lesions close to the great vessels. The laser destruction of tissue including tissue protein denaturation, coagulative necrosis, tissue liquefaction, vaporization, and carbonization occurs instantaneously. Although part of the heat is removed by blood flow, the size of the affected area is still around 12–15 mm in diameter when 3 W of power were used for 10 min.

In the treatment of lesions close to vessels, the impact of laser ablation on vessel should also be considered. If the laser fiber is too close to the vessel, heat will produce tissue damage in the form of denaturation of proteins within the vessel wall, stimulation of inflammation, and vein shrinkage and thrombosis [31]. If the tip of the laser catheter is in contact with the vessel wall, high-energy absorption can lead to tissue vaporization, resulting in vessel perforation. Temperatures above 100 °C will cause tissue vaporization but produce gas bubbles which do not increase the risk of embolization [31]. Leakage into a vessel can cause endothelial damage followed with thrombosis. We did not observe these vessel complications in current study perhaps because the laser fiber was maintained at least 0.5-cm distant from the adjacent vessel.

A clinical sign of retroperitoneal lymph node or hepatic portal metastasis is abdominal pain due to compression or infiltration of celiac plexus [32]. Relief or partial relief of the pain is one of the important treatment aims. Although analgesia with anesthetics can alleviate the pain, it has no effect on tumor control. From the current data, the pain score decreased significantly. Possible explanations include local control of the lymph nodes which resulted in direct pain palliation and the thermal effects caused by laser ablation blocked nerves near the lymph nodes.

There are some limitations to the current study. The number of patients was very small, and the follow-up periods were short. Furthermore, only partial responses were achieved in the study. We think a large study is required and perhaps a randomized control trial or a prospective cohort study will be necessary to confirm these results. Various parameters should be studied to establish the relationship between laser parameters, fiber number, fiber distance to vessels, distance between adjacent fibers, lymph node sizes, and treatment results in the future.

## Conclusions

Laser ablation of the metastatic retroperitoneal lymph nodes and hepatic portal lymph nodes benefits the treatment of unresectable malignancies.

## Abbreviations

AFP, alpha fetoprotein; CA19-9, carbohydrate antigen 19-9; CEUS, contrast enhanced ultrasound; CCA, cholangiocarcinoma; CR, complete response; HCC, hepatocellular carcinoma; LRR, Local response rate; LP, lymph node progression; NC, no change; Nd:YAG, neodymium-doped yttrium aluminum garnet; ORR , overall response rate; PR, partial response
